# Prognostic characterization of OAS1/OAS2/OAS3/OASL in breast cancer

**DOI:** 10.1186/s12885-020-07034-6

**Published:** 2020-06-19

**Authors:** Yujie Zhang, Chaoran Yu

**Affiliations:** 1 Department of Gastrointestinal Surgery Center and Department of Laboratory Medicine, Tongji Hospital, Tongji Medical College, Huazhong University of Science and Technology, Wuhan, 430030 Hubei China; 2 Fudan University Shanghai Cancer Center, Fudan University, Shanghai, 200025 P.R. China; 3 Department of Oncology, Shanghai Medical College, Fudan University, Shanghai, 200025 P.R. China

**Keywords:** OAS, Breast cancer, KM-plotter, Prognosis

## Abstract

**Background:**

Prognostic biomarkers remain a focus in breast cancer during last decades. More reliable predictors to adequately characterize the prognosis of breast cancer are essential. The 2′-5′-oligoadenylate synthetases (OAS), composing of OAS1, OAS2, OAS3, and OAS-like (OASL), are interferon (IFN)-induced antiviral enzymes, with their prognostic roles remain to be characterized.

**Methods:**

Prognostic values of OAS family members were assessed by multiple public available resources.

**Results:**

High mRNA expression of OAS1 and OAS3 were correlated with worse prognosis for all breast cancer patients, whereas OAS2 was associated with favorable prognosis. The prognostic values of OAS family in different clinicopathologic subtypes were also characterized. In DNA methylation level, cg12560128 in OAS2, cg06800840 and cg26328872 in OASL showed significant prognostic values. The mRNA expression of OAS members signature in high/low risk overall survival groups was opposite to the high/low risk recurrence free survival groups. Neutrophil cell exhibited highest correlation with all OAS members in tumor immune infiltrating estimation.

**Conclusions:**

This study provided new insight into the prognostic roles of OAS in breast cancer with potential mechanistic values.

## Background

Breast cancer is one of the top three most common cancers and the most common malignancy for women worldwide [1]. In 2017, approximately 250,000 new cases of invasive breast cancer were diagnosed in women, and more than 40,000 women were predicted to die from breast cancer in the US [2]. According to the 2018 global cancer statistics, breast cancer remains the most commonly diagnosed cancer and the leading cause of cancer death (11.6% of the global cancer deaths) in female patients [3]. In fact, approximately 0.5 million deaths annually are from metastatic breast cancer [4].

Although its mortality has decreased in both North America and the European Union, breast cancer remains a major challenge with increasing incidence in Asia and Africa [1, 2]. Specifically, both the incidence and mortality of breast cancer are rising in Africa. However, high-quality clinical data related to breast cancer are largely lacking in low- and middle-income countries. Significant challenges remain regarding effective therapeutic strategies [5]. In addition, by 2012, approximately 53% of new breast cancer cases occurred in less developed countries rather than more developed countries [6]. Although the incidence rates remain high in more developed countries, this epidemiological distribution is now shifting and serves as a major health issue in Asia and Africa [6].

Based on systemic and multidisciplinary treatment, improved outcomes have been achieved in some cases of breast cancer, while the overall outcomes remain unsatisfactory [1, 7]. Nonetheless, the insightful clues contributed by basic studies remain far from adequate for the clinical translation of prognostic indicators. Therefore, finding reliable biomarkers has been urgent in breast cancer.

The 2′-5′ oligoadenylate synthetase (OAS) family consists of antiviral enzymes induced by interferon and is responsible for the destabilization of virus-derived dsRNA with RNase L function [8]. The OAS family, including OAS1, OAS2, OAS3 and OASL, features a 5 exon -coded structure with various splice variants [8–10]. The OAS family has been well characterized in enzymatic functions [8]. However, the prognostic value of the OAS family has rarely been studied.

Previously, we published bioinformatics research focusing on trastuzumab-resistant gastric cancer. Interestingly, OAS1, OAS2, OAS3 and OASL were all identified as hub genes. Given that OAS1–3 and OASL both belong to the OAS family, it is noteworthy to systematically explore whether OAS family members could be prognostic indicators in breast cancer. Of note, a recent study reported potential inhibitors of the OAS family, further highlighting the multiple functions of OAS in diseases [11]. Overall, it is essential to design an original study that fully characterizes the prognostic value of OAS family members in breast cancer.

The technical progress of bioinformatics and publicly available gene expression profiles provide feasible and reliable approaches for the characterization of the prognostic value of the OAS family in breast cancer. This study focused on the prognostic value of the OAS family in breast cancer using multiple bioinformatics strategies.

## Methods

### Kaplan-Meier plotter

Kaplan-Meier (KM) plotter was used to determine the prognostic value of OAS family expression in breast cancer (http://kmplot.com/analysis/index.php?p=background) [12, 13]. The KM plotter was initially established as a survival analysis platform for gene expression. Currently, KM plotter has been developed into a meta-analysis biomarker database across 20 cancer types [12–14]. As for breast cancer, this genomic platform has incorporated a list of gene expression profiles, including E-MTAB-365(*n* = 537), E-TABM-43 (*n* = 37), GSE11121 (*n* = 200), GSE12093 (*n* = 136), GSE12276 (*n* = 204), GSE1456 (*n* = 159), GSE16391 (*n* = 55), GSE16446 (*n* = 120), GSE16716 (*n* = 47), GSE17705 (*n* = 196), GSE17907 (*n* = 54), GSE18728 (*n* = 61), GSE19615 (*n* = 115), GSE20194 (*n* = 45), GSE20271(*n* = 96), GSE2034 (*n* = 286), GSE20685 (*n* = 3327), GSE21653 (*n* = 240), GSE2603 (*n* = 99), GSE26971 (*n* = 276), GSE2990 (*n* = 102), GSE31448 (*n* = 71), GSE31519 (*n* = 67), GSE32646 (n = 115), GSE3494 (*n* = 251), GSE37946 (*n* = 41), GSE41998 (*n* = 279), GSE42568 (*n* = 121), GSE45255 (*n* = 139), GSE4611 (*n* = 153), GSE5327 (*n* = 58), GSE6532 (*n* = 82), GSE7390 (*n* = 198) and GSE9195 (*n* = 77) for prognostic analysis. In fact, the included profiles were standardized prior to analysis. In general, OAS1, OAS3 and OASL expression was found in 1402 patients, while OAS2 expression was found in 626 patients. The expression of OAS members was divided into high and low expression groups using the optimal cut-off values algorithm. Given that the original design of this study was to fully characterize the prognostic values of OAS family members across various subtypes in breast cancer as well as general groups, we further investigated their prognostic values in subsets. Stratified analyses of clinical factors, including pathological grades, lymph node (LN) status, and intrinsic subtype, were also performed for survival evaluation.

In brief, gene expression profiles (microarray) from a variety of studies were selected. The cutoff value of OAS family members was determined by an auto-select-best-cutoff algorithm embedded in the KM plotter website. This algorithm calculated all possible cutoff values between the lower and upper quartiles and determined an optimal cutoff with the most significan t statistical value. Overall survival was selected as the endpoint. Statistically, a *p*-value< 0.05 (log rank p-value) was considered a significant Hazard ratio (HR), and the corresponding 95% confidence intervals (95% CI) was also displayed.

### The mRNA expression of OAS members in the cancer genome atlas (TCGA) database

The mRNA expression of OAS family members was analyzed in the breast cancer in the Gene Expression Profiling Interactive Analysis Platform (GEPIA, http://gepia.cancer-pku.cn/index.html) [15].

### Prognostic roles of the DNA methylation of OAS members

The DNA methylation results of the OAS family and corresponding survival analysis were performed via the MethSurv platform, an integrated online tool for TCGA methylation analysis (https://biit.cs.ut.ee/methsurv/) [16]. Breast invasive carcinoma (BRCA) was selected for the OAS cancer type.

### Prognostic role of the OAS member signature

The prognostic values of the OAS signature for both overall survival (OS) and recurrence-free survival (RFS) were investigated by the SurvExpress platform (http://bioinformatica.mty.itesm.mx:8080/Biomatec/SurvivaX.jsp) using the meta-base with 1901 breast cancer patients (overall survival). The SurvExpress platform is an integrated survival evaluation resource for multiple cancer types with advanced statistical strategies [17]. The high-risk and low-risk groups were divided by the risk score algorithm embedded in the platform [17].

### Tumor immunological features of OAS members

All immune infiltrate cells (macrophages, neutrophils, dendritic cells, B cells, CD4+ T cells, and CD8+ T cells) were selected for correlation with OAS members based on the Tumor Immune Estimation Resource (TIMER) (https://cistrome.shinyapps.io/timer/) [18]. TIMER is an online tool for tumor immune cell evaluation based on the references from TCGA. The Spearman correlation was corrected by tumor purity [18]. Statistically, a *p*-value< 0.05 was considered significant.

## Results

### Prognostic values of OAS family members in all breast cancer patients

**Fig. 1:**
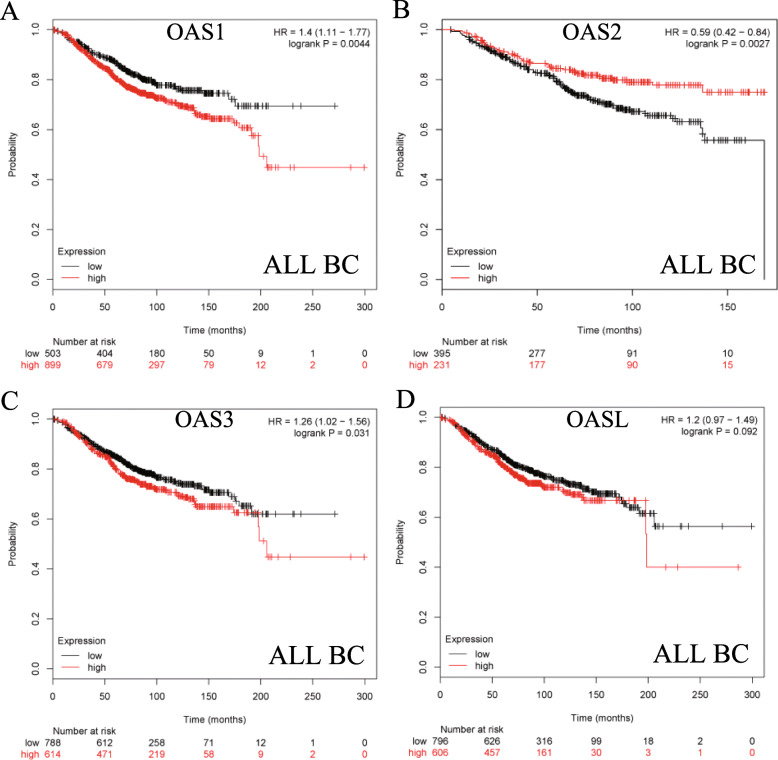
The prognostic values of the mRNA expression of OAS family in KM plotter (www.kmplot.com). Survival curves of OAS1 (**a**) **(**the desired Affymetrix IDs is valid:202869_at), OAS2 (**b**) (Affymetrix IDs: 228607_at), OAS3 (**c**) (Affymetrix IDs: 218400_at), and OASL (**d**) **(**Affymetrix IDs: 205660_at) are plotted for all breast cancer patients

The prognostic values of the mRNA expression of OAS family members were explored via the KM plotter. The mRNA expression of OAS1, OAS3 and OASL was found in a total of 1402 patients, while OAS2 was found in 626 patients (Additional file 3: Table S1). OAS1, OAS2, and OAS3 were significantly associated with prognosis for all breast cancer patients (Fig. 1a-d). High mRNA expression of OAS1 and OAS3 was correlated with worse prognosis (HR =1.4, 95%CI: 1.11–1.77, *p* = 0.0044 and HR = 1.26, 95% CI: 1.02–1.56, *p* = 0.031) (Fig. 1c and d). High mRNA expression of OAS2 was significantly associated with better OS (HR = 0.59, 95% CI: 0.42–0.84, *p* = 0.0027) (Fig. 1b). However, OASL was not correlated with OS (HR = 1.2, 95% CI: 0.97–1.49, *p* = 0.092) (Fig. 1d).

### Prognostic values of OAS family members in breast cancer subtypes

**Fig. 2:**
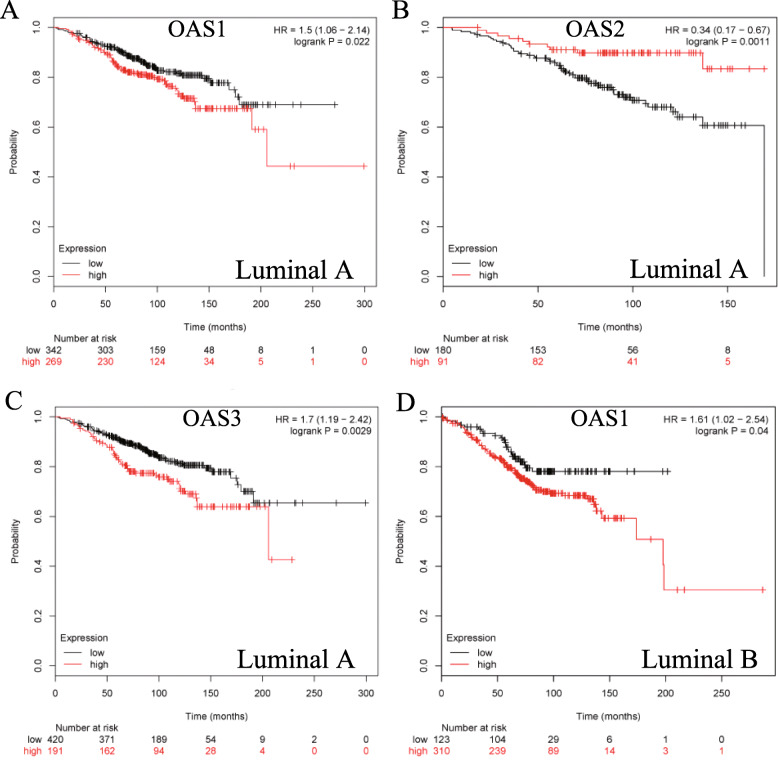
Survival curves (OS) of OAS1 (**a**), OAS2 (**b**), and OAS3 (**c**) are plotted for luminal A type breast cancer patients. Survival curve (OS) of OAS1 (**d**) is plotted for luminal B type breast cancer patients

**Fig. 3:**
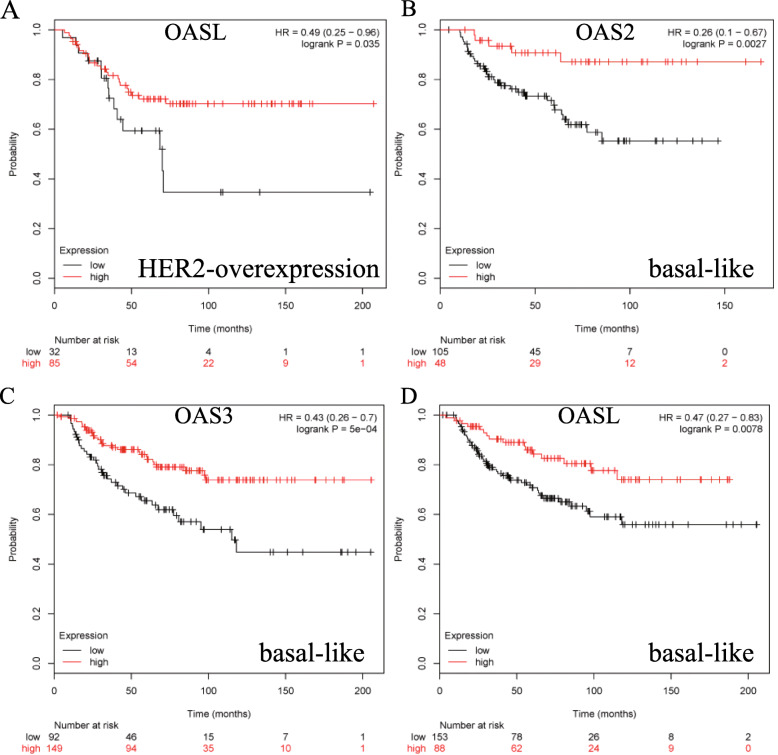
Survival curve (OS) of OASL (**a**) is plotted for HER2-overexpression breast cancer patients. Survival curves (OS) of OAS2 (**b**), OAS3 (**c**), and OASL (**d**) are plotted for basal-like breast cancer patients

Next, the prognostic values of OAS members were explored in breast cancer with different intrinsic subtypes, including the luminal A, luminal B, HER2 overexpression and basal-like subtypes. High mRNA expression of OAS1 (HR = 1.5, 95%CI: 1.06–2.14, *p* = 0.022) and OAS3 (HR = 1.7, 95%CI: 1.19–2.42, *p* = 0.0029) was significantly associated with worse OS in luminal A type breast cancer patients (Fig. 2a, c). High mRNA expression of OAS2 (HR = 0.34, 95%CI: 0.17–0.67, *p* = 0.0011) was correlated with better OS in luminal A type cancers (Fig. 2b), whereas OASL was not related to prognosis in luminal A breast cancer (Additional file 1: Figure S1A). In luminal B type breast cancer, high mRNA expression of OAS1 was correlated with worse OS (HR = 1.61, 95%CI: 1.02–2.54, *p* = 0.04) (Fig. 2d). The remaining OAS family members were not correlated with prognosis in luminal B type breast cancer (Additional file 1: Figure S1B-D). In HER2-overexpressing breast cancer patients, high mRNA expression of OASL was correlated with favorable OS (HR = 0.49, 95%CI: 0.25–0.96, *p* = 0.035) (Fig. 3a). The remaining members of the OAS family in HER2-overexpressing breast cancer patients were not correlated with prognosis (Additional file 2: Figure S2A-C). High mRNA expression of OAS2 (HR = 0.26, 95%CI: 0.1–0.67, *p* = 0.0027), OAS3 (HR = 0.43, 95%CI: 0.26–0.7, *p* < 0.001), and OASL (HR = 0.47, 95%CI: 0.27–0.83, *p* = 0.0078) was correlated with better OS in basal-like breast cancer (Fig. 3b-d). However, OAS1 was only modestly associated with better OS but without a significant difference (Additional file 2: Figure S2D).

### Prognostic values of OAS family members in breast cancer patients with clinicopathological subtypes

The prognostic values of OAS family members in association with pathological grades, and lymph node status were explored. High mRNA expression of OAS1 was significantly associated with worse OS in grade 1 breast cancer (HR = 2.71, 95%CI: 1.11–6.61, *p* = 0.022), while OAS2 analysis showed the opposite results, as high OAS2 mRNA expression was significantly correlated with a better OS prognosis (HR = 0, 95%CI: 0-lnf, *p* = 0.0071) (Table 1). High mRNA expression of OAS1 (HR = 2.01, 95%CI: 1.26–3.18, *p* = 0.0026), OAS3 (HR = 1.72, 95%CI: 1.13–2.64, *p* = 0.011) and OASL (HR = 1.8, 95%CI: 1.17–2.77, *p* = 0.0066) was significantly associated with worse OS in grade 2 breast cancer. High mRNA expression of OAS3 (HR = 0.65, 95%CI: 0.47–0.9, *p* = 0.0088) and OASL (HR = 0.62, 95%CI: 0.44–0.86, *p* = 0.0035) was found to be correlated with better OS in grade 3 breast cancer (Table 1). High mRNA expression of OAS1 (HR = 1.8, 95%CI: 1.23–2.62, p = 0.002) and OAS3 (HR = 1.69, 95%CI: 1.15–2.46, *p* = 0.0065) was correlated with worse survival in lymph node-negative breast cancer patients (Table 2). High mRNA expression of OAS2 (HR = 0.33, 95%CI: 0.13–0.83, *p* = 0.014) was associated with better OS in lymph node-negative breast cancer patients (Table 2). High mRNA expression of OAS3 (HR = 0.59, 95%CI: 0.37–0.94, *p* = 0.024) was associated with better OS in lymph node-positive breast cancer patients (Table 2).

**Table 1:** Correlation of OAS with different pathological grade status of breast cancer patients

OAS family	Affymetrix IDs	grades	HR (95% CI)	***P***-value
OAS1	202869_at	I	2.71 (1.11–6.61)	0.022*
		II	2.01 (1.26–3.18)	0.0026*
		III	0.73 (0.52–1.01)	0.059
OAS2	228607_at	I	0 (0-lnf)	0.0071*
		II	0.33 (0.09–1.21)	0.079
		III	0.7 (0.39–1.28)	0.24
OAS3	218400_at	I	2.22 (0.8–6.18)	0.12
		II	1.72 (1.13–2.64)	0.011*
		III	0.65 (0.47–0.9)	0.0088*
OASL	205660_at	I	2 (0.79–5.08)	0.14
		II	1.8 (1.17–2.77)	0.0066*
		III	0.62 (0.44–0.86)	0.0035*

*: *P* < 0.05.

**Table 2:** Correlation of OAS family members with different lymph node status of breast cancer patients

OAS family	Affymetrix IDs	Lymph node status	HR (95% CI)	***P***-value
OAS1	202869_at	negative	1.8 (1.23–2.62)	0.002*
		positive	1.3 (0.86–1.95)	0.22
OAS2	228607_at	negative	0.33 (0.13–0.83)	0.014*
		positive	0.73 (0.43–1.24)	0.24
OAS3	218400_at	negative	1.69 (1.15–2.46)	0.0065*
		positive	0.59 (0.37–0.94)	0.024*
OASL	205660_at	negative	1.41 (0.94–2.11)	0.092
		positive	0.75 (0.51–1.11)	0.15

*: *P* < 0.05.

### Analysis of the mRNA expression of OAS family in TCGA

**Fig. 4:**
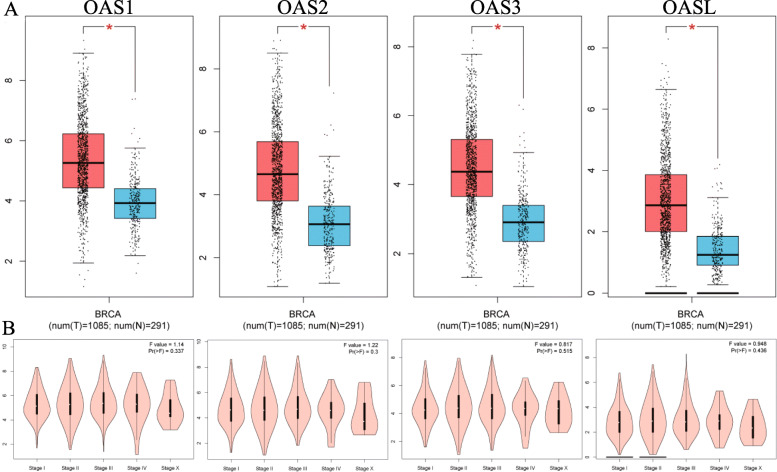
The mRNA expression of OAS family members in Breast Cancer (BRCA) of TCGA. **a** The mRNA expression of OAS family between tumor and normal tissues; (**b**) The stage-specifically mRNA expression of OAS family

No significant stage-specific expression was found for OAS members in BRCA. However, all OAS family members featured significant upregulation in tumors compared to normal tissues (Fig. 4a-b).

### Prognostic values of OAS signature

**Fig. 5:**
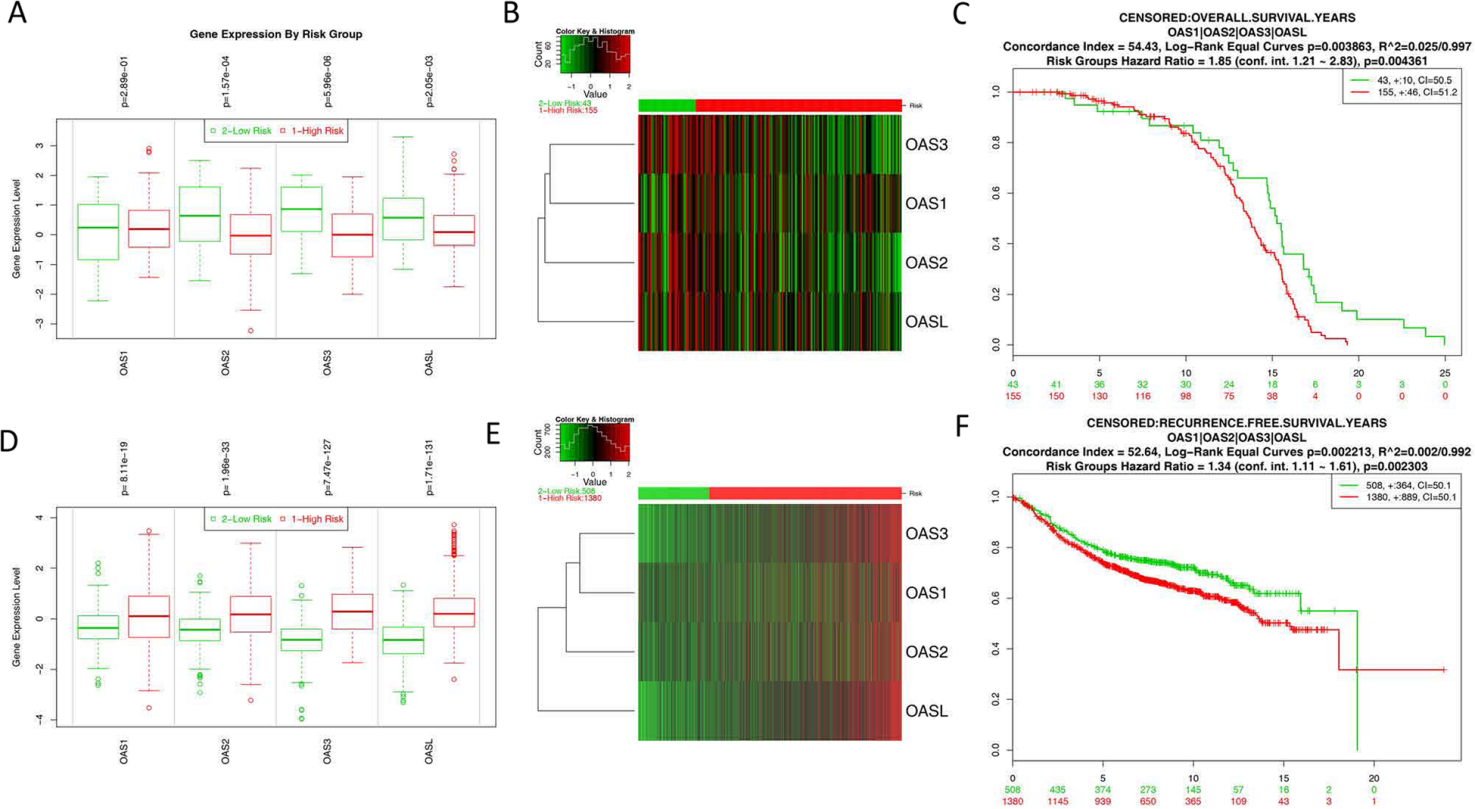
The prognostic values of OAS members signature in BRCA via SurvExpress platform. **a** The mRNA expression of OAS family between high and low risk groups (overall survival, OS); (**b**) the mRNA expression of OAS family displayed in heat map (OS); (**c**) the prognostic results of OAS family between high and low risk groups (OS); (**d**) The mRNA expression of OAS family between high and low risk groups (recurrence free survival, RFS); (**e**) the mRNA expression of OAS family displayed in heat map (RFS); (**f**) the prognostic results of OAS family between high and low risk groups (RFS)

Given the increasing focus on the prognostic value of gene signatures, the OAS signature was also input for prognostic analysis in SurvExpress. For OS, the mRNA expression of OAS members, except OAS1, was higher in the low-risk group than in the high-risk group. The low-risk group displayed a favorable OS outcome compared with the high-risk group (Fig. 5a-c). Intriguingly, for RFS, the mRNA expression of OAS members was higher in the high-risk group than in the low-risk group (Fig. 5d-e). The low-risk group displayed a favorable RFS outcome compared with the high-risk group (Fig. 5f).

### The prognostic values of DNA methylation of OAS members

**Fig. 6:**
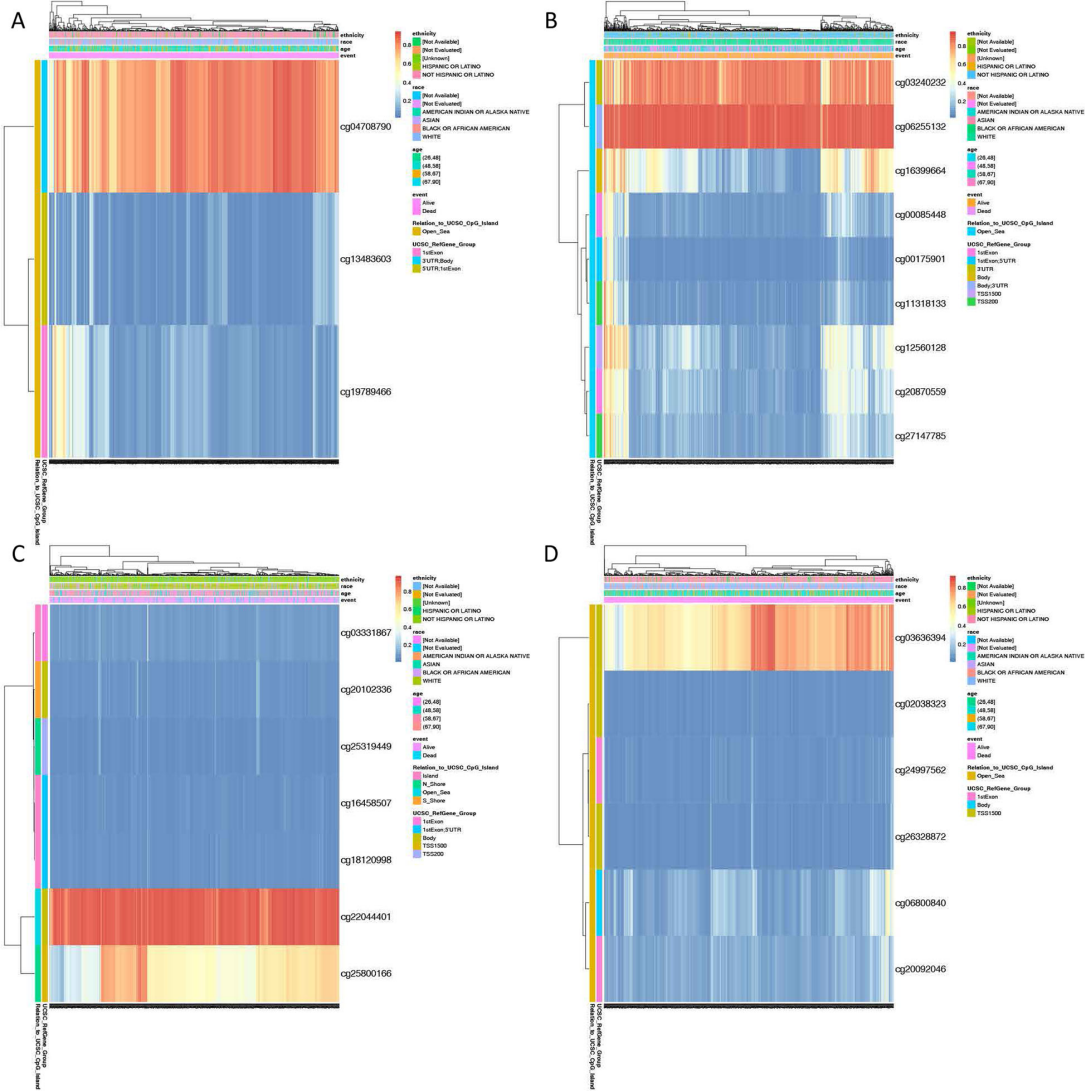
The DNA methylation of OAS family members in BRCA of TCGA. **a** The DNA methylation of OAS1; (**b**) the DNA methylation of OAS2; (**c**) the DNA methylation of OAS3; (**d**) the DNA methylation of OASL

**Fig. 7:**
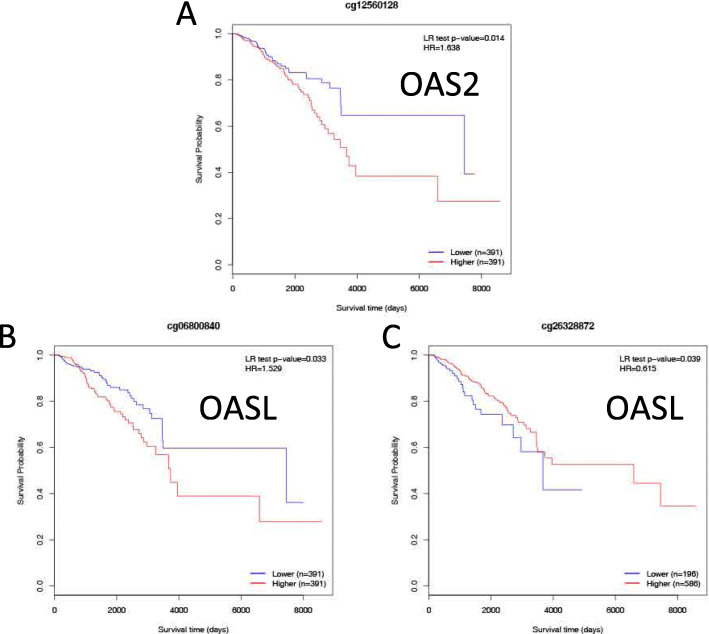
The significant prognostic values of DNA methylation of OAS family members. **a** The prognostic value of DNA methylation cg12560128 in OAS2; (**b**) the prognostic value of DNA methylation cg06800840 in OASL; (**c**) the prognostic value of DNA methylation cg26328872 in OASL

All the DNA methylation results of the OAS members are displayed (Fig. 6a-d). Moreover, the DNA methylation patterns of OAS members with significant prognostic value were also identified, including cg12560128 in OAS2 and cg06800840 and cg26328872 in OASL (Fig. 7a-c).

### The correlation between OAS members and tumor immune infiltrating cells

**Fig. 8:**
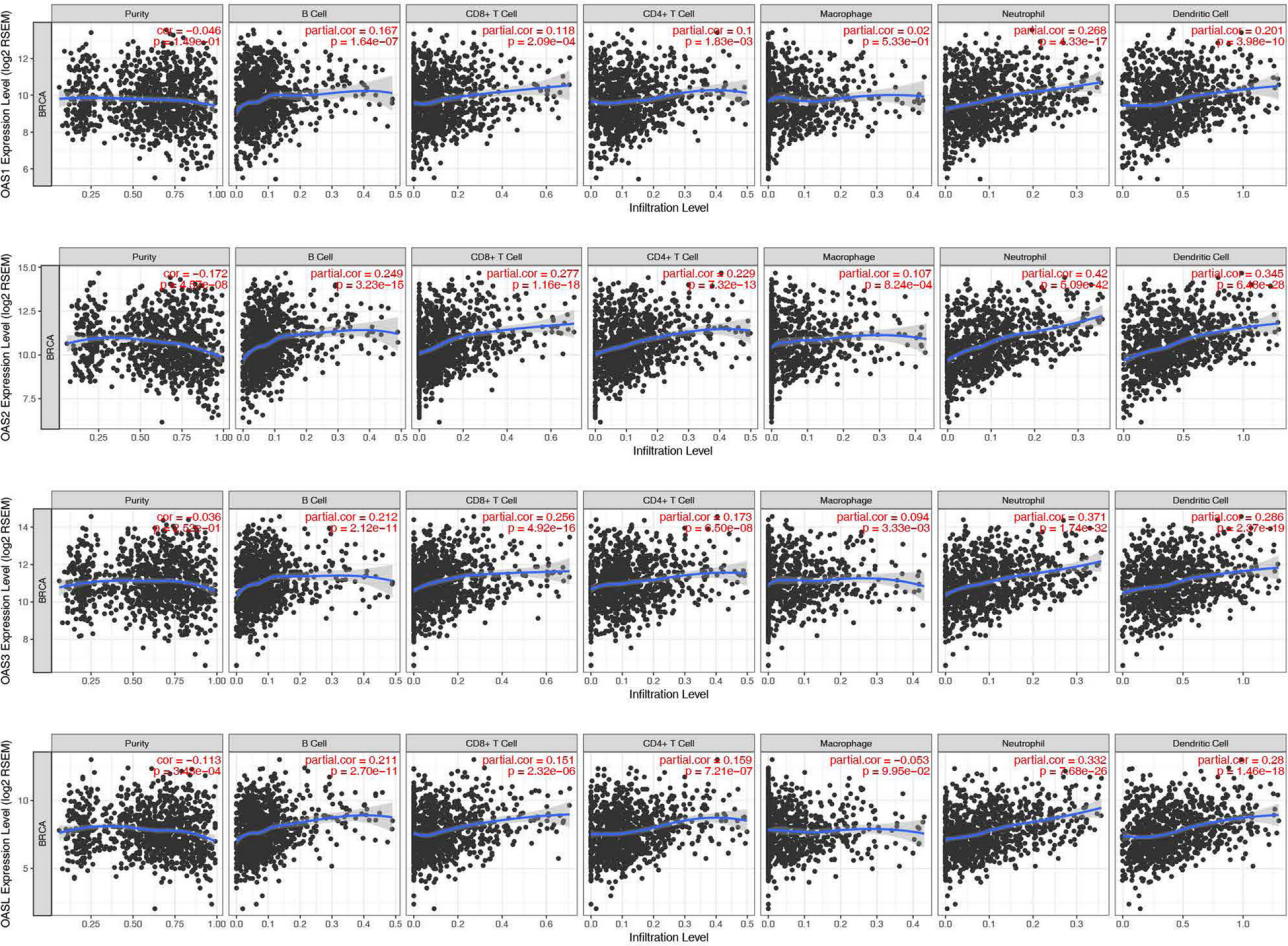
The correlation of OAS members and tumor infiltrating immune cells via Tumor Immune Estimation Resource (TIMER) (https://cistrome.shinyapps.io/timer/)

Next, the potential correlation of OAS members and tumor immune cells (B cells, CD4+ T cells, CD8+ T cells, neutrophils, macrophages and dendritic cells) was estimated based on TCGA data. Interestingly, neutrophils exhibited the highest correlation with OAS members (cor = 0.268 in OAS1; cor = 0.42 in OAS2; cor = 0.371 in OAS3; cor = 0.332 in OASL), highlighting the key role of OAS members associated with neutrophils in tumor immune infiltrating cells (Fig. 8).

## Discussion

In summary, we found that high mRNA expression of OAS1 and OAS3 was correlated with worse prognosis in all breast cancer patients. Moreover, a distinct prognostic signature of OAS family members was established. This study provided insightful clues that OAS family members may be used as novel prognostic biomarkers in breast cancer.

Previously, OAS family members have been found to be involved in a variety of diseases, including infections, autoimmune disorders and cancer. Their functions include antiviral modulation, apoptosis control, cell growth, differentiation and gene regulation [19–26]. In 1986, Liu et al. reported a potential correlation between OAS activity and tumor growth in human mammary tumors [27]. Marino et al. demonstrated that OAS1 was able to affect cell migration [28]. Moreover, OASL was considerably related to cancer proliferation [26]. Our data highlighted the prognostic values of OAS members in mRNA expression and DNA methylation, as well as the gene signature, enabling multilevel insights for these prognostic predictors. High mRNA expression of OAS1 and OAS3 was correlated with worse prognosis, while high mRNA expression of OAS2 was associated with better outcomes in all breast cancer patients. Moreover, our study further demonstrated that high OAS1 expression predicts poor survival in breast cancer patients, especially in subgroups with LN-negative, luminal A, luminal B, grade I and grade II tumors.

**Fig. 9:**
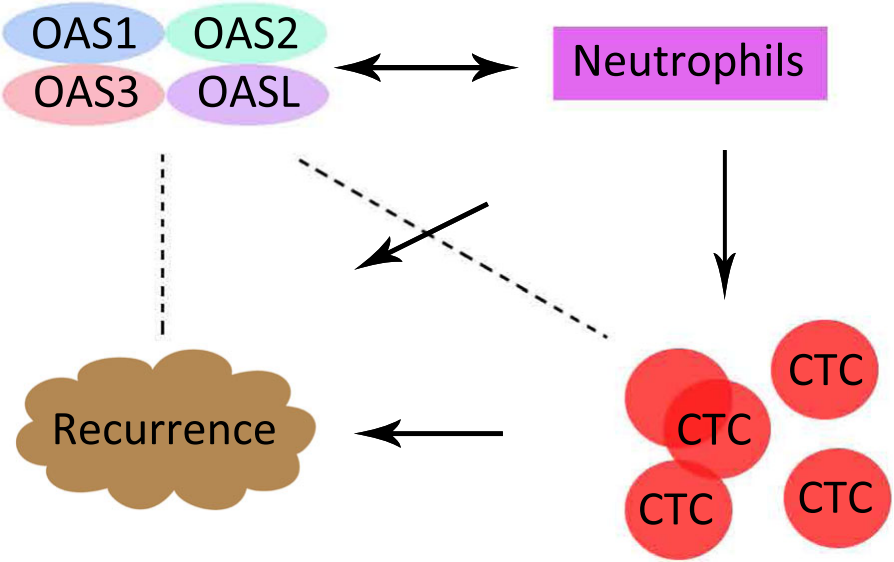
The hypotheses of recurrence-associated mechanism of OAS members. ‘↓’ indicate promoted effects; “← → “indicates correlation from our analysis; “---” indicate hypothetic regulation mechanism

Neutrophils are involved in the metastatic/recurrent potential of circulating tumor cells (CTCs) in colorectal cancer by the CTC-neutrophil cluster pattern [29]. Specifically, CTCs clustered with neutrophils displayed a significant enrichment of cell cycle and DNA replication programs compared to CTCs that were not associated with neutrophils [29]. Moreover, neutrophils also contribute to tumor metastasis through multiple pathways, including PI3K-Akt, chemokines and cytokines [30]. Interestingly, neutrophils were the top tumor immune infiltrating cell type associated with OAS members according to this study. Notably, the mRNA expression of the OAS member signature in the high−/low-risk OS groups was opposite that in the high−/low-risk RFS groups, indicating a potential recurrence-associated role of OAS members (Fig. 9).

Although mechanistic insights into the associations between the OAS family and several types of diseases have been increasingly investigated, their roles in drug targets remain largely unexplored. Our previous research indicated that OAS1, OAS2, OAS3 and OASL were all identified as hub genes from the protein-protein interaction network of differentially expressed genes between the trastuzumab-resistant gastric cancer and control groups [11]. Interestingly, a recent study reported 37 molecules as inhibitors of the OAS family, particularly interacting with the Asp75, Asp77, Gln229 and Tyr230 in OAS1–3, further supporting their potential as drug targets [31]. These potential candidates exert competitive inhibition over ATP binding sites without a significant impact on enzymatic activation [31].

The present study had several limitations. First, the findings revealed in this study warrant further experimental or clinical validation. Second, the current findings only identified noticeable features associated with the prognosis of OAS with only hypothetically mechanistic exploration.

## Conclusion

This study provided new insight into the prognostic roles of OAS in breast cancer with potential target values.

## Supplementary information

**Additional file 1: Figure S1.** Prognostic values of OAS family in breast cancer (luminal A & luminal B) (A-D).

**Additional file 2: Figure S2.** Prognostic values of OAS family in breast cancer (HER2-overexpression & basal-like) (A-D).

**Additional file 3: Table S1.** ER/PR/HER2/lymph node/TP53 status, histological grade and intrinsic subtypes of included cases.

## Data Availability

All data generated or analysed during this study are included in this published article [and its supplementary information files].

## Abbreviations

CI: Confidential intervals
GEO: Gene Expression Omnibus
HR: Hazard ratio
HER2: Epidermal growth factor receptor 2
LN: Lymph node
OS: Overall survival
OAS1: 2′-5′-Oligoadenylate synthetase 1
OAS2: 2′-5′-Oligoadenylate synthetase 2
OAS3: 2′-5′-Oligoadenylate synthetase 3
OASL: 2′-5′-Oligoadenylate synthetase-like
CTCs: Circulating tumor cells
TIMER: Tumor Immune Estimation Response
OS: Overall survival
RFS: Recurrence-free survival
BRCA: Breast invasive carcinoma
GEPIA: Gene Expression Profiling Interactive Analysis Platform
