# Supplementary figures and images for: Prognostic characterization of OAS1/OAS2/OAS3/OASL in breast cancer
Source: BMC Cancer. 2020 Jun 19;20:575. doi: 10.1186/s12885-020-07034-6 (PMC7304174; doi:10.1186/s12885-020-07034-6)

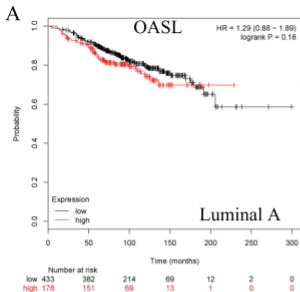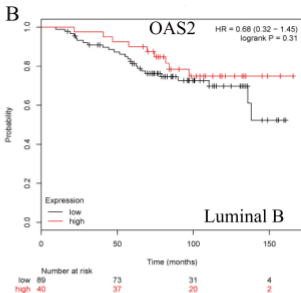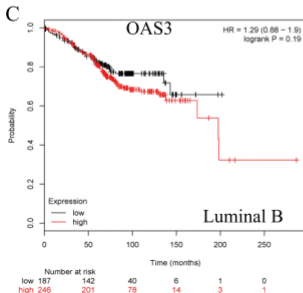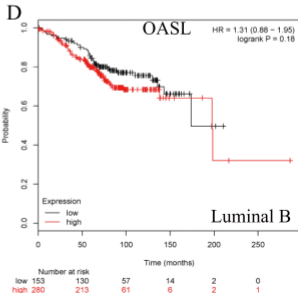

Supplement: Supplementary file 1 — Additional file 1: Figure S1. Prognostic values of OAS family in breast cancer (luminal A & luminal B) (A-D). [file 12885_2020_7034_MOESM1_ESM.pdf]

A

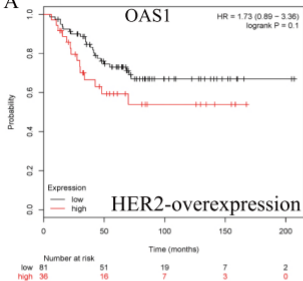

B

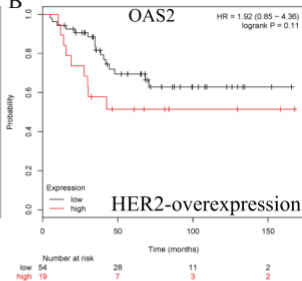

C

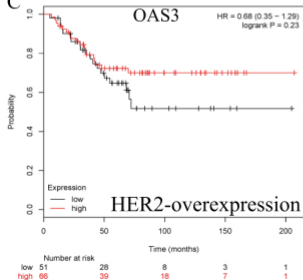

D

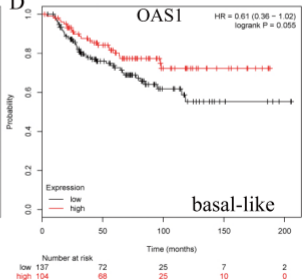

Supplement: Supplementary file 2 — Additional file 2: Figure S2. Prognostic values of OAS family in breast cancer (HER2-overexpression & basal-like) (A-D). [file 12885_2020_7034_MOESM2_ESM.pdf]
