# Supplementary material for: Prognostic characterization of OAS1/OAS2/OAS3/OASL in breast cancer
Source: BMC Cancer. 2020 Jun 19;20:575. doi: 10.1186/s12885-020-07034-6 (PMC7304174; doi:10.1186/s12885-020-07034-6)
Supplement: Supplementary file 3 — Additional file 3: Table S1. ER/PR/HER2/lymph node/TP53 status, histological grade and intrinsic subtypes of included cases. [file 12885_2020_7034_MOESM3_ESM.docx]

**Supplementary table 1**: ER/PR/HER2/lymph node/TP53 status, histological grade and intrinsic subtypes of included cases.

|  | OAS1 | OAS2 | OAS3 | OASL |
| --- | --- | --- | --- | --- |
| **All cases** | 1402 | 626 | 1402 | 1402 |
|  |  |  |  |  |
| **ER status** |  |  |  |  |
| ER positive | 548 | 109 | 548 | 548 |
| ER negative | 251 | 79 | 251 | 251 |
|  |  |  |  |  |
| **PR status** |  |  |  |  |
| PR positive | 83 | 0 | 83 | 83 |
| PR negative | 89 | 0 | 89 | 89 |
|  |  |  |  |  |
| **HER2 status** |  |  |  |  |
| HER2 positive | 129 | 26 | 129 | 129 |
| HER2 negative | 130 | 62 | 130 | 130 |
|  |  |  |  |  |
| **Lymph node status** |  |  |  |  |
| Lymph node positive | 313 | 177 | 313 | 313 |
| Lymph node negative | 594 | 122 | 594 | 594 |
|  |  |  |  |  |
| **TP53 status** |  |  |  |  |
| mutated | 111 | 56 | 111 | 111 |
| wild typre | 187 | 6 | 187 | 187 |
|  |  |  |  |  |
| **Grade** |  |  |  |  |
| Grade 1 | 161 | 26 | 161 | 161 |
| Grade 2 | 387 | 64 | 387 | 387 |
| Grade 3 | 503 | 204 | 503 | 503 |
|  |  |  |  |  |
| **Intrinsic subtype** |  |  |  |  |
| basal | 241 | 153 | 241 | 241 |
| luminal A | 611 | 271 | 611 | 611 |
| luminal B | 433 | 129 | 433 | 433 |
| HER2+ | 117 | 73 | 117 | 117 |
